# Endometrioid adenocarcinoma with simultaneous endocervical and intestinal-type mucinous differentiation: report of a rare phenomenon and the immunohistochemical profile

**DOI:** 10.1186/1746-1596-8-128

**Published:** 2013-08-02

**Authors:** Rebecca Buell-Gutbrod, C James Sung, W Dwayne Lawrence, M Ruhul Quddus

**Affiliations:** 1 Department of Pathology and Laboratory Medicine, Women & Infants Hospital and the Warren Alpert Medical School of Brown University, 101 Dudley Street, Providence, RI 02905, USA

**Keywords:** Endometrium, Endometrioid adenocarcinoma, Intestinal differentiation, Goblet cells

## Abstract

**Abstract:**

Intestinal differentiation in the endometrium is rare with only case reports in the international literature. We describe a case of simultaneous endocervical and intestinal-type mucinous differentiation with goblet cells arising in a FIGO grade 1 endometrioid adenocarcinoma. The patient had no involvement of the myometrium, cervix, or extra-uterine sites. There were no intestinal metaplastic changes of the endocervical canal. The etiology of this change is unknown, although recent reports suggest an association with hyperestrogenism.

**Virtual slides:**

The virtual slides for this article can be found here: http://www.diagnosticpathology.diagnomx.eu/vs/1209512176931698

## Introduction

Endocervical-type mucinous differentiation in endometrioid adenocarcinoma is not uncommon. However, intestinal differentiation in the endometrium is rare with only case reports in the international literature. These included two cases associated with endometrial carcinoma where the intestinal differentiation was uncovered upon application of special stains [1] and two cases associated with an endometrial polyp and endometrial hyperplasia [2]. Reports of rare carcinoma of the endometrium, their immunohistocheamical staining patterns and tissue reaction to endometrial carcinoma are available in the literature [3-5]. We present a case of simultaneous intestinal differentiation with goblet cells and endocervical-type mucinous differentiation in a FIGO grade 1 endometrioid adenocarcinoma.

## Case presentation

The patient is a 55 year old gravida 2, para 2 with a chief complaint of postmenopausal bleeding. Her past medical history is significant for morbid obesity (BMI 40), type II diabetes, hypertension, and asthma. CT scan showed heterogeneity of the uterus and bilateral hydrosalpinx, with no evidence of metastatic disease. The patient underwent an endometrial biopsy and a subsequent robotic assisted total laparoscopic hysterectomy and bilateral salpingo-oophorectomy. The post-surgical course was unremarkable and the patient was discharged on post-op day 3.

## Pathological findings

Endometrial biopsy revealed a well differentiated endometrioid adenocarcinoma, FIGO grade 1, arising in a background of complex atypical hyperplasia. On hysterectomy, the uterus measured 6.0 × 5.5 × 4.5 cm uterus with a smooth glistening serosal surface and an attached cervix. The specimen was opened to reveal a 1.5 × 1.5 cm intrauterine mass occupying the posterior aspect of the uterine fundus. The remainder of the 3.9 × 3.8 cm endometrium was pink-tan. The myometrium was grossly uninvolved by tumor. The endocervical canal was unremarkable. Bilateral adnexae consisted of white bosselated ovaries and previously ligated fallopian tube with fimbriated ends. The endometrium was submitted in its entirety.

**Figure 1:**
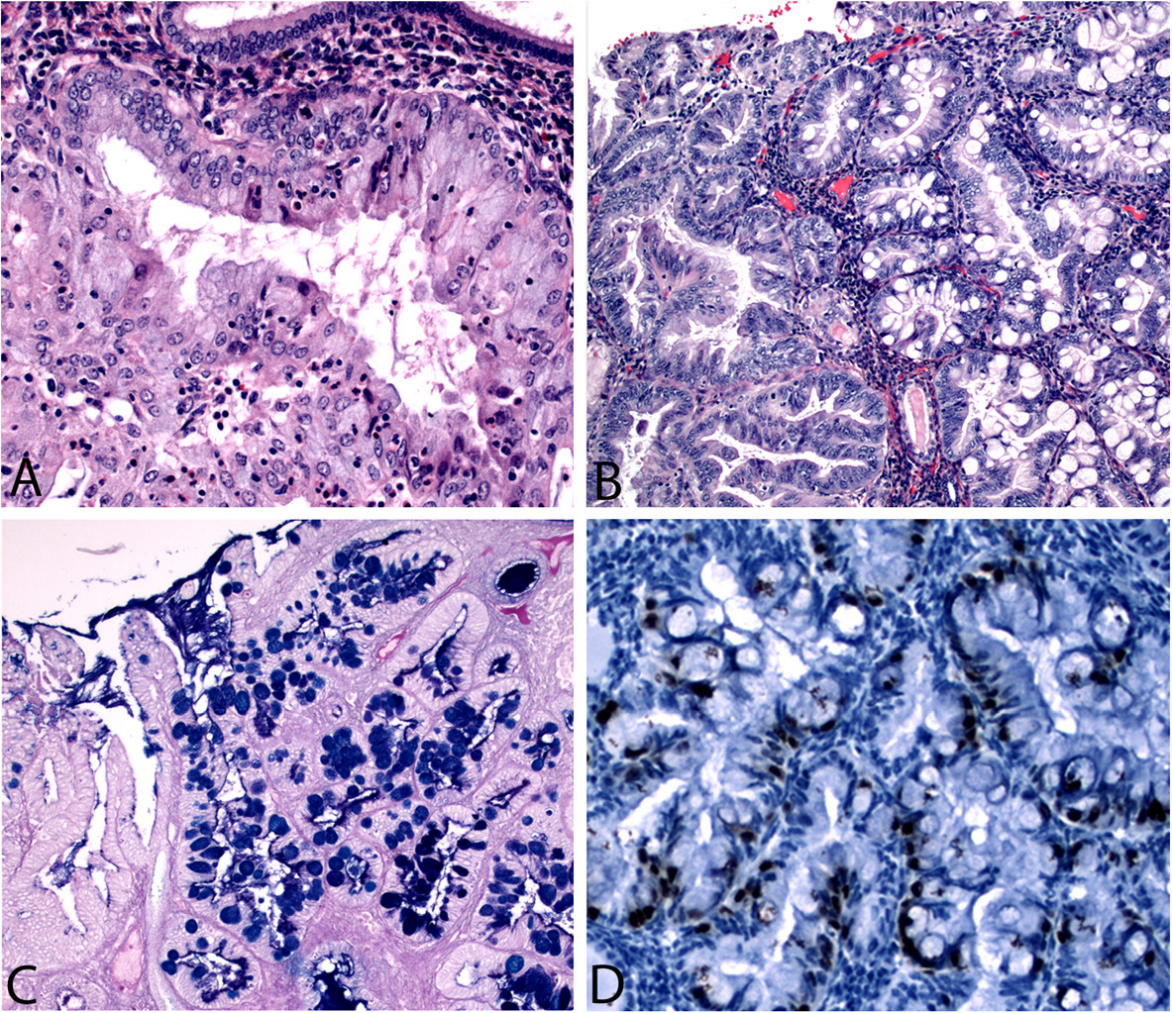
**Endometrioid adenocarcinoma with endocervical (A) and intestinal type (B) mucinous differentiation.** Classic endometrioid adenocarcinoma is seen in the left side of panel **B** adjacent to the intestinal-type metaplasia. AB-PAS highlights goblet cells with deep blue staining **(C)**. CDX-2 is positive in the intestinal type cells **(D)**.

Microscopically, the tumor consisted primarily of an endometrioid adenocarcinoma of usual type which focally showed endocervical-type mucinous differentiation (Figure 1A). Endocervical-type mucinous differentiation is characterized by tall columnar mucin filled cells with basally located nuclei. Two adjacent microscopic foci also showed readily visible intestinal-type mucinous differentiation with basally located hyperchromatic nuclei and goblet cells (Figure 1B). There was involvement of superficial adenomyosis by tumor, but no true myometrial invasion. As in the prior biopsy, a background of complex atypical endometrial hyperplasia was noted. The lower uterine segment and cervix were free of tumor and so was lymph-vascular invasion. No intestinal-type metaplastic change was noted in the endocervical canal. Bilateral fallopian tubes showed only benign changes and a small serous adenofibroma was discovered in the left ovary.

Special stains and immunohistochemical (IHC) analysis was employed to further characterize the cells of interest. Alcian blue-perioidic acid-schiff stain (AB-PAS) stains acid mucins such as those in goblet cells blue while the neutral mucins of endocervical-type cells are pink as in the classic PAS stain. AB-PAS stain in this case confirms the morphologic impression of goblet cells by staining the acid mucin a deep blue color while the endocervical-type mucinous cells show faint pink staining (Figure 1C). IHC of the areas with intestinal differentiation revealed Cytokeratin (CK) 7 positivity, while CK20 was negative. CDX2 (Figure 1D) and CEA were focally positive in the cells of interest, while synaptophysin and chromogranin were completely negative (Table 1).

**Table 1:** Immunohistochemical profile of intestinal metaplasia in G1 EMCA

**Antibody**	**Antibody information**	**Results**
Cytokeratin 7	M; OV-TL 12/30; Dako	Positive
Cytokeratin 20	M; Ks20.8; Dako	Negative
CDX2	M; DAK-CDX-2; Dako	Focally positive
Carcinoembryonic Antigen (CEA)	M; II-7; Dako	Focally positive
Synaptophysin	M; SY38; Dako	Negative
Chromogranin	P; Rabbit; Dako	Negative

*M:* Monoclonal, *P:* Polyclonal.

## Discussion and conclusions

Mucinous differentiation in endometrioid adenocarcinomas is not uncommon and pure mucinous adenocarcinomas of the endometrium comprise 1-10% of all endometrioid adenocarcinomas. Most mucin producing tumors of the endometrium are well differentiated with endocervical-type mucin producing cells characterized by tall columnar cells with basally located nuclei. However, the presence of intestinal differentiation, and of goblet cells in particular, within the endometrium is rare. The largest series of endometrial goblet cells differentiation in the world literature reports only two cases, which were identified with the assistance of special stains [1]. Two cases of intestinal metaplasia, one in an endometrial polyp and one in association with complex hyperplasia without atypia, have recently been reported [2]. Neither was associated with endometrial carcinoma, in contrasts to ours. One was associated with concurrent intestinal metaplasia of the endocervical canal. Two cases of endometrial adenocarcinomas with signet ring cells have been reported in the literature [6] and an additional case of mucinous adenocarcinoma of the endometrium was found to have a gastric phenotype on immunohistochemistry mimicking a minimal deviation adenocarcinoma of the cervix [7]. As in this case, all were associated with more typical appearing endometrioid adenocarcinoma. Enteric-type mucin has been identified in endometrial adenocarcinomas, a finding which verifies the ability of the Mullerian epithelium to undergo metaplasia along an intestinal lineage [8]. No cases in that series, however, showed morphologic intestinal differentiation and there was no correlation with tumor grade.

The presence of goblet cell within the endometrium raises the legitimate question for metastasis. Bland well-differentiated intestinal-type mucinous epithelium with goblet cell in the endometrium, endocervix, and fallopian tubes, has been shown to be metastatic lesions from gastrointestinal primaries [9]. Clinical evaluation for an occult primary gastrointestinal lesion as well as careful gross and histologic exam is necessary. Cytokeratins are useful in this setting as metaplastic Mullerian epithelium retains CK7 positivity, as in our case. In this current case, the patient had no clinical gastrointestinal lesion. The gross exam was that of a classic non-invasive carcinoma arising from endometrial. No involvement of the adnexa, serosa, or the myometrium by the tumor was noted and the cells with intestinal differentiation were intimately associated and readily visible with the endometrioid carcinoma.

Intestinal-type metaplasia is more common within the endocervix, with reports of cases in non-neoplastic and endocervical adenocarcinoma [1,10]. Intestinal metaplasia in the endocervix is associated with both adenocarcinoma in-situ and endocervical adenocarcinoma and the presence of intestinal-type cells on cervical biopsy warrants further workup.

The etiology of intestinal-type differentiation within the endometrium is unclear. The two recently reported cases associated with non-neoplastic lesions were associated with probable hyperestrogenism [2], a finding replicated in this patient who was obese and had a background of endometrial hyperplasia. This is in contrast to the proposed mechanism of endocervical-type mucinous metaplasia which has been linked to use of tamoxifen and exogenous progestin [11,12].

With the addition of our case, the number of cases of intestinal-type differentiation with goblet cells in the endometrium is increased to five, with two cases being benign and two cases associated with adenocarcinoma only detected with special stains [1,2]. This entity is extremely rare, this being the first case encountered in our sub-specialty practice and consult service.

The clinical implication of finding intestinal-type differentiation in endometrial carcinoma, as in our case report, remains in the fact that the pathologists and clinicians needed to be aware that such differentiation may occur in primary endometrial carcinoma and that the finding of intestinal-type mucinous differentiation in endometrial carcinoma should also warrant careful clinico-pathologic work-up to exclude a metastatic carcinoma from the gastro-intestinal tract.

## Consent

The patient has given written consent for the use of the images and case presentation for educational and scientific purposes provided the unique personal identification is not revealed.

## Competing interests

The authors declare no competing financial interest. All the authors have actively participated in the diagnosis process or manuscript writing.

## Authors’ contributions

RBG is the Stuart Lauchlan Fellow in Gynecologic and Breast Pathology and has written up the case report and MRQ is the attending Pathologist on the case. CJS and WDL were involved in interpreting the special stains. All authors read and approved the final manuscript.
